# Liposomal bupivacaine in thoracic surgery: a comprehensive review of current evidence and clinical implications

**DOI:** 10.3389/fmed.2026.1917255

**Published:** 2026-09-11

**Authors:** Liuchun Huang, Jing He, Jiaping Wei, Xiuming Huang, Xianglan Chen, Qi Yang, Yonglong Zhong

**Affiliations:** 1 School of Nursing, Guangxi University of Chinese Medicine, Nanning, Guangxi Zhuang Autonomous Region, China; 2 Department of Thoracic Surgery, The People’s Hospital of Guangxi Zhuang Autonomous Region, Guangxi Academy of Medical Sciences, Nanning, Guangxi Zhuang Autonomous Region, China; 3 Nursing Department, The People’s Hospital of Guangxi Zhuang Autonomous Region, Guangxi Academy of Medical Sciences, Nanning, Guangxi Zhuang Autonomous Region, China

**Keywords:** liposomal bupivacaine, opioid-sparing, postoperative analgesia, regional anesthesia, thoracic surgery

## Abstract

Liposomal bupivacaine (LB) is a prolonged-release formulation designed to provide postoperative analgesia for up to 72-96 h after a single injection. This comprehensive review evaluates the current evidence on LB in thoracic surgery, focusing on acute pain control, opioid consumption, hospital length of stay (LOS), functional recovery, comparative effectiveness across regional techniques, integration into Enhanced Recovery After Surgery (ERAS) protocols, chronic postsurgical pain (CPSP) reduction, and safety/cost considerations. The core critical finding of this review is that although meta-analyses show statistically significant reductions in pain scores and opioid consumption with LB, the clinical significance of these reductions is questionable—for example, the opioid-sparing effect at 24 h (-1.83 MME) is far below the threshold generally considered clinically meaningful (10–15 MME). Results vary by block type; benefits are more consistent with serratus anterior, erector spinae, and paravertebral blocks, while intercostal nerve block data are mixed. LB appears most effective when incorporated into ERAS protocols, although the independent contribution of LB within these multimodal pathways remains difficult to isolate. Emerging evidence suggests LB may reduce CPSP incidence by 30–58%, but these findings are preliminary and require confirmation in larger studies with longer follow-up. Safety profiles are favorable, with peak plasma concentrations well below toxicity thresholds. Methodological limitations of the existing evidence include small sample sizes, lack of blinding, and and heterogeneity across studies. Future large-scale RCTs with long-term follow-up are warranted.

## Introduction

Thoracic surgery has undergone substantial evolution over the past few decades, with video-assisted thoracoscopic surgery (VATS) and robotic-assisted procedures becoming increasingly common. Nevertheless, managing postoperative pain remains a significant clinical challenge. Inadequate pain control after thoracic operations can result in impaired lung function, delayed mobilization, higher opioid use, and longer hospital stays (1, 2). The pain after thoracic surgery is multifactorial—stemming from chest wall incisions, intercostal nerve irritation, and pleural inflammation—necessitating effective, long-lasting pain relief strategies.

Traditional approaches for postoperative pain in thoracic surgery include thoracic epidural analgesia (TEA), systemic opioids, and regional nerve blocks using conventional local anesthetics. TEA has long been considered the gold standard, but it is associated with side effects such as hypotension, urinary retention, and technical difficulties (3). Several studies have compared LB with TEA as an alternative approach (4, 5). Regular local anesthetics such as bupivacaine hydrochloride and ropivacaine are effective but have a short duration of action—typically 6–8 h—necessitating repeated doses or continuous infusions. For example, Marciniak et al. (6) and Zhang et al. (7) used conventional bupivacaine or ropivacaine as controls in their studies.

Liposomal bupivacaine (LB) is a novel formulation in which bupivacaine is encased in multivesicular liposomes, designed to release the drug slowly over a prolonged period—reportedly up to 72–96 h after a single injection (2, 8). This extended action offers the theoretical benefit of continuous pain relief without the need for a catheter or repeated injections. LB has attracted considerable interest in the thoracic surgery field, with many studies examining its efficacy across different regional analgesia techniques and in various clinical contexts.

This review aims to provide a comprehensive appraisal of the current evidence on the use of liposomal bupivacaine in thoracic surgery. We examine its effectiveness for acute pain control, its impact on opioid use, its effects on hospital stay length and functional recovery, its comparative performance across different regional analgesia techniques, its role in Enhanced Recovery After Surgery (ERAS) protocols, its potential to reduce chronic postsurgical pain, and relevant safety, pharmacokinetic, and cost considerations.

## Methods

This review was conducted as a comprehensive narrative review. A systematic literature search was performed in PubMed (May 12, 2026) using search terms combining “liposomal bupivacaine” (and related terms) with “thoracic surgery” (and related procedural terms). Eligible studies included randomized controlled trials, observational studies, systematic reviews, and meta-analyses evaluating LB for postoperative analgesia in adult thoracic surgery. Reference lists of included articles were also screened. Given the heterogeneity in study designs, block techniques, and outcome measures, findings were synthesized narratively rather than through meta-analysis. A PRISMA flow diagram was not generated, and the review was not prospectively registered, consistent with its narrative nature.

## Overall efficacy of liposomal bupivacaine for postoperative analgesia

A comprehensive meta-analysis pooling nine RCTs with 915 patients demonstrated that LB reduced pain scores at 24 h by a mean difference of -0.99 and at 48 h by -0.42 compared with non-liposomal local anesthetics (9). Similarly, a separate meta-analysis confirmed that LB was associated with lower resting VAS scores at 24, 48, and 72 h and also reduced movement-evoked pain at all three time points (10). These pooled estimates suggest that LB provides measurable analgesic benefit in the early postoperative period.

In contrast, another meta-analysis that included both observational studies and RCTs reported no significant differences in postoperative pain scores between LB and non-liposomal local anesthetics in thoracic surgery patients (2). A randomized trial comparing combined pectoralis and serratus anterior plane blocks with LB plus bupivacaine versus bupivacaine alone also found no meaningful improvement in the Overall Benefit of Analgesia Score during the first three postoperative days (6). These discrepancies might be attributable to differences in block techniques, dosing regimens, and patient populations across studies. For instance, the meta-analysis that favored LB predominantly included studies using paravertebral or erector spinae blocks, whereas the negative meta-analysis had a higher proportion of intercostal nerve block studies. Variability in the timing of pain assessment and the use of rescue analgesics also likely contributed to the divergent results (11).

Several individual trials have provided more granular evidence. For SAPB, an RCT demonstrated that ultrasound-guided LB injection led to significantly lower pain scores at rest and with movement from 12 to 72 h compared with ropivacaine (7). For ICNB, a prospective RCT showed that LB produced significantly lower VAS scores at rest and during exercise at all six measured time points from 6 to 72 h (12). Taken together, these findings support the notion that LB can offer sustained analgesia when administered via appropriate regional techniques, although the magnitude of benefit varies.

Pooled analysis from a meta-analysis showed that LB reduced opioid consumption at 24 h by 1.83 MME, at 48 h by 2.22 MME, and at 72 h by 1.73 MME compared with control groups (9). Another meta-analysis reported reductions in morphine consumption of 2.68 mg within 24 h and 8.76 mg within 72 h (10). While these differences reached statistical significance, their clinical relevance is questionable. Although the MCID for pain scores has been defined (13), a well-established MCID for postoperative opioid consumption in thoracic surgery has not been formally defined. Nevertheless, reductions of less than 10-15 MME over 24 h are generally considered unlikely to be clinically meaningful (14). An absolute reduction of 1.83 MME (equivalent to roughly 1.8 mg of oral morphine) is far below this threshold, suggesting that the opioid-sparing effect observed in these pooled analyses may not translate into meaningful patient benefits. Nevertheless, the direction of effect is consistent, and some individual trials reported larger reductions.

Individual RCTs have reported larger opioid-sparing effects in specific settings. For example, one trial found that PCA demand frequency was significantly lower in the LB group (median 11 vs. 30 presses) and patient satisfaction scores were higher (12). Another study observed reduced opioid consumption after uniportal thoracoscopy with LB ICNB (15). A third trial demonstrated that LB for TPVB significantly lowered 72-hour opioid consumption by a mean difference of 181.4 mg compared with ropivacaine (16).

However, some studies haven't found significant opioid-sparing benefits. A randomized trial in minimally invasive lobectomy found no difference in in-hospital morphine equivalents between bupivacaine and LB groups (42.7 mg vs. 48 mg) (17). Similarly, a retrospective cohort study reported that cumulative opioid consumption was similar between LB and control groups (198 mg vs. 195 mg intravenous morphine equivalents) (18). These negative findings might reflect differences in baseline opioid use, surgical techniques, or other pain management regimens.

A retrospective analysis reported a significantly shorter median LOS in patients receiving LB compared to standard bupivacaine (1.35 days vs. 2.45 days) (19). In a study of 302 minimally invasive lobectomy patients, LOS was nearly a full day shorter in the LB group than in the standard bupivacaine group (34.8 vs. 56.5 h) (20). Another retrospective analysis found that across all thoracic surgery types, LOS was lower in the LB group (median 4 vs. 5 days) (21). On the other hand, several randomized trials didn't find significant differences in LOS. Two randomized trials reported similar LOS between bupivacaine and LB groups (17, 18). The meta-analysis by Gong et al. concluded that LB was not better than non-liposomal local anesthetics at reducing LOS (2).

Functional recovery outcomes have also varied. One study found that 93% of patients receiving LB were able to ambulate within 24 h after surgery, compared to 65% in the control group (19). Another trial showed that LB was associated with shorter time to first ambulation and first defecation (16). A third study noted earlier first flatus, mobilization, and urinary catheter removal with LB (7). These findings suggest that LB might help patients recover function sooner, possibly through improved pain control and reduced opioid-related side effects.

The contradictory findings between these meta-analyses highlight a key point: the efficacy of LB is highly dependent on the specific block technique and cannot be generalized. Importantly, no head-to-head RCTs directly comparing LB efficacy across different block techniques currently exist, representing an important gap in the evidence base (22).

The very small effect size for opioid reduction raises an important question: in the context of thoracic ERAS protocols that already incorporate multimodal analgesia (including acetaminophen, NSAIDs, dexmedetomidine, etc.), does the addition of LB provide meaningful incremental benefit? This question cannot be definitively answered by existing meta-analyses, as most primary studies allowed rescue analgesics but did not standardize non-opioid analgesic regimens, and the independent contribution of LB within multimodal protocols has not been isolated in factorial-design trials (Table 1).

**Table 1:** Comparison of Key study results.

Author (year)	Study type	Sample size	Primary block technique	Pain score (MD)	Opioid consumption (MD)	CPSP effect	Evidence level^a^
Jin & Wang (2026) (9)	Meta-analysis	915	Paravertebral/ESPB	24h: −0.99; 48h: −0.42	24h: −1.83 MME; 48h: −2.22 MME; 72h: −1.73 MME	Not reported	Moderate
Hu et al. (2026) (10)	Meta-analysis	930	Peripheral nerve blocks	Significantly lower at 24h, 48h, 72h	24h: −2.68 mg; 72h: −8.76 mg	Not reported	Moderate
Gong et al. (2024) (2)	Meta-analysis	1,086	Mixed (more ICNB)	No significant difference	No significant difference	Not reported	Low
Shang et al. (2026) (32)	RCT	146	TPVB	Lower pain AUC over 72h	Significantly reduced vs. bupivacaine	58% reduction (28.6% vs. 48.6%)	High
Yan et al. (2026) (42)	Retrospective cohort	1,325	ICNB	Reduced at 24h and 48h	Significantly reduced	42% reduction (OR 0.68)	Low-moderate

a Evidence level: High = multicenter double-blind RCT; Moderate = single-center RCT or meta-analysis with limitations; Low = observational study or meta-analysis with poor methodological quality.

## Comparative effectiveness across different regional analgesia techniques

### Intercostal nerve block

Intercostal nerve block is one of the most widely used regional analgesia techniques in thoracic surgery, and the use of liposomal bupivacaine for ICNB has been studied extensively. A systematic review and meta-analysis that included two RCTs and eight observational studies found that LB for ICNB did not reduce opioid use at 24 h, 48 h, or for the total hospital stay compared to standard bupivacaine (23). Pain scores were also not significantly different between groups at 24 and 48 h, and there was no difference in LOS (23). These findings suggest that the benefits of LB for ICNB may be limited. That said, several individual studies have reported positive outcomes with LB for ICNB. A prospective RCT demonstrated that LB provided sustained and effective postoperative analgesia for 72 h while significantly reducing opioid consumption and supplemental analgesic requirements compared to conventional bupivacaine (12). Another randomized trial reported that LB was safe and effective for ICNB, providing significant pain relief for uniportal thoracoscopic surgery (15). A retrospective study found that patients receiving LB for ICNB consumed fewer analgesics than those receiving bupivacaine/epinephrine, with statistically significant differences observed from 24–36 h and from 60–72 h postoperatively (24). A comparative study of LB ICNB placed under direct vision versus thoracic epidural analgesia showed that LB was associated with lower median pain scores at 24 and 48 h, reduced narcotic requirements at 48 h, fewer postoperative complications, and shorter median hospital LOS (25). Notably, a retrospective analysis found that LB reduced opioid use across all surgery types, with the benefit most pronounced in patients undergoing robotic-assisted thoracic surgery (21). This suggests that the efficacy of LB for ICNB may depend on the specific surgical approach.

### Serratus anterior plane block

The serratus anterior plane block has become a popular regional analgesia technique for thoracic surgery. An RCT comparing LB to ropivacaine for SAPB in patients undergoing VATS lobectomy found that the LB group had significantly lower pain scores at rest and with movement at multiple time points from 12 to 72 h postoperatively (7). Perioperative sufentanil use and postoperative flurbiprofen axetil use were significantly reduced in the LB group. Moreover, the LB group had earlier first flatus, mobilization, and urinary catheter removal, shorter ICU stay, lower incidence of postoperative nausea and vomiting, and lower hospitalization costs (7). A separate trial compared continuous SAPB with bupivacaine to single-shot SAPB with LB and found that the single LB injection provided superior early postoperative recovery quality, as measured by QoR-15 scores at 24 and 48 h (26). The LB group also had a significantly reduced incidence of local complications such as swelling and pain (26). These findings suggest that LB for SAPB can offer effective, long-lasting analgesia and may have advantages over both conventional local anesthetics and continuous infusion methods.

### Erector spinae plane block

The erector spinae plane block has emerged as a promising regional analgesia technique for thoracic surgery. A multicenter RCT involving 272 patients undergoing VATS lung resection compared LB to bupivacaine hydrochloride for preoperative ultrasound-guided ESPB (27). The 72-hour cumulative rest pain was significantly lower in the LB group (205.0 vs. 232.5), although this difference did not reach the predefined MCID. However, pain scores at 24 and 32 h, both at rest and during activity, did achieve the clinically important difference of 1 point. LB was associated with lower opioid use and higher recovery quality (27). Another study evaluated the immunomodulatory and analgesic effects of LB compared to ropivacaine for ESPB in thoracoscopic lung cancer surgery (28). The LB group exhibited significantly reduced inflammatory responses with lower levels of IL-6, TNF-*α*, and CRP at 12–72 h postoperatively. Immunoglobulin levels were better preserved in the LB group, and analgesia outcomes were superior with lower pain scores at 36 and 72 h (28). These findings suggest that LB for ESPB might offer immunoprotective benefits in addition to analgesic effects, although the clinical significance of these immunological changes requires further validation. A further trial reported that patients receiving LB for ESPB had lower rest pain scores during the first 48 h, higher QoR-15 scores, reduced postoperative sufentanil consumption, and improved pulmonary function indices compared to those receiving conventional bupivacaine (29). Time to first ambulation was shorter, and time to first request for analgesia was longer in the LB group (29).

### Thoracic paravertebral block

Thoracic paravertebral block is a well-established regional analgesia technique for thoracic surgery. A randomized controlled study comparing LB to plain bupivacaine for TPVB in patients undergoing VATS found that while opioid consumption did not differ between groups at 48 h, the LB group had higher QoR-15 scores at 24 h and a longer time to first analgesia request (585.8 vs. 315.3 min) (30). The area under the curve for pain scores at rest was significantly lower in the LB group (30). Another RCT demonstrated that ultrasound-guided TPVB with LB significantly reduced 72-hour opioid consumption compared to ropivacaine (mean difference -181.4 mg) and was associated with shorter time to first ambulation and defecation (16). A separate randomized study reported that LB for TPVB provided superior pain control compared to ropivacaine, with significantly lower cough pain scores at 24, 48, and 72 h, as well as a lower incidence of vomiting and dizziness (31). A prospective double-blind RCT involving 146 patients undergoing VATS found that LB for TPVB significantly reduced the area under the curve for pain scores at rest and during movement over the first 72 h compared to bupivacaine hydrochloride (32). The LB group also had a 58% reduction in CPSP incidence at 1 and 3 months (28.6% vs. 48.6%) (32). A further trial compared LB to bupivacaine combined with dexamethasone for TPVB and found that LB significantly prolonged the duration of analgesia (1,160.4 vs. 743.1 min), representing a 56% extension (33). The LB group had lower resting and cough pain scores on the first postoperative day and shorter times to first ambulation and first flatus (33).

## Summary of comparative evidence

The evidence for ICNB is the most heterogeneous, with positive results primarily from retrospective studies whereas RCTs have largely been negative—suggesting that observational studies may be prone to selection bias (34). The evidence for SAPB and TPVB is more consistent, but these studies have mostly come from single centers or the same research group, lacking multi-center independent validation. ESPB, as a newer technique, has limited available studies, and the clinical significance of its immunomodulatory effects requires further validation (Table 2). Therefore, based on current evidence, it is not possible to definitively determine which block technique combined with LB has an absolute advantage; selection should be based on surgical type, patient factors, and institutional experience (35).

**Table 2:** Evidence summary and critical appraisal by block technique.

Block technique	Number of studies	Consistency	Effect size (pain)	Effect size (opioid)	Major limitations	Recommendation grade^a^
ICNB	High	Low	Small to moderate	Inconsistent	Positive mainly from retrospective studies; negative RCTs	Weak
SAPB	Moderate	High	Moderate	Moderate	Small sample sizes, mostly single-center	Moderate
ESPB	Moderate	Moderate	Small to moderate	Moderate	Limited studies, mostly single-center	Moderate
TPVB	High	High	Moderate	Moderate to large	Some studies not blinded	Strong

a Recommendation grade: Strong = evidence supports routine use; Moderate = may be considered, more evidence needed; Weak = insufficient or conflicting evidence, not recommended for routine use.

## LB in enhanced recovery after surgery protocols and reduction of chronic postsurgical pain

### Enhanced recovery after surgery

Integrating liposomal bupivacaine into Enhanced Recovery After Surgery (ERAS) protocols for thoracic surgery has attracted considerable interest, since multimodal pain management is a cornerstone of these pathways. A cohort study comparing three groups of patients undergoing lobectomies (control receiving neither LB nor ERAS, LB alone, and LB as part of an ERAS protocol) found a statistically significant reduction in opioid use across all groups, with the greatest reduction observed in the ERAS/LB cohort (17 OME) compared to the control group (43 OME) and the LB-alone cohort (30.5 OME) (36). This finding highlights the potential synergistic effect of combining LB with comprehensive ERAS protocols, although the study design does not allow isolation of LB's independent contribution from other ERAS components. An evaluation of an ERAS protocol incorporating LB for robotic thoracic surgery showed that protocol optimization—including replacement of saline diluent with 0.25% bupivacaine and switching tramadol to as-needed administration—resulted in a significant reduction of in-hospital and post-discharge opioid requirements without affecting acute pain levels (37). The median schedule II opioids prescribed was zero in the optimized protocol group (37). Implementation of an ERAS protocol with multimodal pain management, including LB infiltration to intercostal spaces and surgical sites, was associated with significant reduction of postoperative pain and opioid requirements in both robotic thoracoscopy and thoracotomy patients (38). Median in-hospital opioid use dropped from 30 to 18.36 MME per day for the robotic thoracoscopy group, and median post-discharge opioids prescribed were drastically reduced from 480.0 to 150.0 MME (38). An optimized ERAS protocol incorporating LB resulted in 66% of patients being discharged without opioids following robotic thoracic surgery (39). Only 3.2% of opioid-free discharge patients became new persistent opioid users compared to 10.8% of patients filling opioid prescriptions after discharge (39). A sustained reduction in discharge opioid prescriptions over a 4-year ERAS program was reported, with the rate declining from 35% to 25% and the amount of opioid prescribed declining from 184 to 94 MME (40). LB management as a component of an active enhanced recovery program was associated with a reduction in major pulmonary complications compared to nonuse (odds ratio 0.63) (41). This finding suggests potential for LB to contribute to improved clinical outcomes beyond pain control when integrated into comprehensive ERAS pathways, but causality cannot be inferred from this observational association.

The most notable finding in ERAS studies is the achievement of “opioid-free discharge” when LB is combined with comprehensive ERAS protocols (39). However, most of these studies used a before-after design, making it difficult to distinguish the independent contribution of LB from other components of the ERAS protocol. Furthermore, “opioid-free discharge” does not equate to “pain-free,” and the studies did not report patient-reported pain satisfaction, representing an important omitted outcome.

### Chronic postsurgical pain

Chronic postsurgical pain (CPSP) is a major complication after thoracic surgery, affecting a substantial proportion of patients and impairing quality of life. A large retrospective cohort study of 1,325 patients undergoing VATS lung resection found that ICNB with LB was associated with a significantly lower incidence of CPSP at 3 months compared to ropivacaine (33.5% vs. 42.3%; adjusted odds ratio 0.68) (42). Patients receiving LB also had reduced pain scores at 24 and 48 h postoperatively. These findings suggest that the prolonged analgesic effect of LB may prevent the transition from acute to chronic pain, although prospective confirmation is needed (42). A prospective double-blind RCT reported that LB for TPVB resulted in a 58% reduction in CPSP incidence at 1 and 3 months postoperatively compared to bupivacaine hydrochloride (28.6% vs. 48.6%) (32). The LB group also demonstrated attenuated inflammatory responses, as indicated by decreased IL-1*β* levels within 48 h and lower CRP levels at 24 h (32). This anti-inflammatory effect may contribute to the reduction in CPSP. Another RCT found that LB for TPVB was associated with lower chronic pain scores at 1-3 months postoperatively compared to ropivacaine (43). However, the LB group had a higher incidence of postoperative nausea and vomiting, possibly related to the prolonged analgesic effect allowing for reduced opioid use but potentially different side effect profiles (43). A protocol for an RCT specifically designed to investigate the role of wound infiltration using LB versus ropivacaine on chronic pain outcomes following VATS has been published (44). The primary outcome is the incidence of CPSP at 3 months, with secondary outcomes including pain at 1 and 6 months postoperatively. This study will provide important prospective data on the long-term analgesic benefits of LB (44).

The mechanism by which LB may reduce CPSP is likely multifactorial. The sustained release of bupivacaine over 72-96 h may provide more effective blockade of nociceptive input during the critical perioperative period, potentially preventing central sensitization and the development of chronic pain states. Additionally, the reduced opioid consumption associated with LB may minimize opioid-induced hyperalgesia, which can contribute to chronic pain development.

The finding that LB reduces CPSP incidence by 30–58% is highly promising but must be interpreted with caution. First, the definition of CPSP and follow-up duration varied across studies; second, the mechanism by which LB reduces CPSP remains unclear—whether it directly blocks central sensitization through prolonged analgesia or indirectly by reducing opioid consumption (45). Existing studies cannot distinguish between these possibilities. Third, the longest follow-up in these studies was 6 months; longer-term CPSP relief effects are unknown.

## Safety, pharmacokinetics, and cost considerations

### Safety

The safety profile of liposomal bupivacaine in thoracic surgery has been evaluated in multiple studies, with generally favorable results. An observational study of the pharmacokinetics of surgeon-performed ICNB with LB for posterolateral thoracotomy found that the median time to peak plasma concentration was 24 h (range 15 min to 48 h) (46). The median peak plasma concentration was 0.6 μg/mL, well below the described toxicity threshold of 2 μg/mL. Serum bupivacaine concentration was undetectable at 96 h in all patients, and no patients developed local anesthetic toxicity (46). However, the generalizability of these pharmacokinetic findings is limited by the small sample size and the specific surgical context, and larger studies are needed to confirm safety across diverse patient populations and block techniques. A propensity score-matched analysis comparing LB ICNB to epidural analgesia in patients undergoing lung resection found no increase in perioperative complications with LB (8). The rates of pulmonary, gastrointestinal, wound, and neurologic complications were similar between groups, and there were actually more cardiovascular complications in the epidural arm, primarily atrial arrhythmias (8). A separate study similarly reported no acute toxicity related to LB in their comparison of posterior ICNB with LB to TEA (4). The safety of LB has been further supported by studies demonstrating no significant differences in adverse events between LB and conventional local anesthetics. One RCT reported no significant differences in chest tube duration, length of hospital stay, or adverse events between LB and ropivacaine groups (15). Another RCT found no significant differences in postoperative nausea and vomiting, pruritus, pulmonary complications, or cardiovascular events between LB and conventional bupivacaine (12).

### Cost-effectiveness

Cost is an important consideration when adopting LB, which is significantly more expensive than conventional local anesthetics. A randomized trial reported that while overall hospital costs were similar between bupivacaine and LB groups ($20,252 vs. $22,775), pharmacy costs for LB were significantly higher ($1,052 vs. $596) (17). However, a different study found that LB was associated with lower total and direct hospital costs ($2,906 and $1,865 respectively) compared to TEA, possibly due to shorter hospital stays and fewer complications (47). Use of LB as part of an ERATS protocol did not increase hospital costs compared to bupivacaine/epinephrine (48). The reduction in opioid requirements and the potential for earlier discharge may offset the higher acquisition cost of LB in some clinical contexts. However, the cost-effectiveness of LB likely varies depending on the specific patient population, surgical approach, and institutional practices, and health care system context, and formal health economic evaluations incorporating indirect costs are needed.

## Quality of evidence and methodological limitations

Most of the studies included in this review have significant methodological limitations. The majority of randomized controlled trials had small sample sizes (typically <100 patients per arm) and lacked blinding of patients or outcome assessors (49). Observational studies, which often reported larger treatment effects, were prone to selection bias and confounding by indication. Among the meta-analyses cited, only two reported performing a risk-of-bias assessment using the Cochrane tool (9, 10), whereas another did not describe any quality appraisal of primary studies (2). Furthermore, none of the meta-analyses formally assessed publication bias using funnel plots or Egger's test, leaving the possibility that small negative trials remain unpublished (45, 50). Consequently, the pooled estimates, particularly for opioid consumption and LOS, should be interpreted with caution. Future systematic reviews should prospectively register their protocols and adhere to PRISMA guidelines to improve transparency (34).

## Critical analysis and future directions

This comprehensive review provides a critical appraisal of the current evidence on liposomal bupivacaine in thoracic surgery. Our main conclusions are as follows: (1) LB shows statistically significant analgesic and opioid-sparing effects with specific block techniques (SAPB, ESPB, TPVB), but the effect sizes are small, and their clinical significance is debatable; (2) the evidence for ICNB is the most mixed, with discrepancies between retrospective studies and RCTs, indicating a need for more high-quality RCTs to clarify its value; (3) LB integrated into ERAS protocols shows potential synergistic effects, but the independent contribution of LB is difficult to isolate given the observational nature of most ERAS studies; (4) the potential of LB to reduce CPSP incidence is a promising but preliminary finding that requires confirmation in larger studies with longer follow-up; and (5) the existing evidence has significant methodological limitations: small sample sizes, lack of blinding, potential for publication bias, and high heterogeneity (34, 45). Therefore, before LB can be routinely recommended in clinical practice, more well-designed, adequately powered, multi-center RCTs are needed, particularly direct comparative studies of different block techniques and studies designed to isolate LB's incremental benefit within multimodal ERAS protocols.

## Methodological limitations of current evidence

Most RCTs have fewer than 100 patients per arm, which may overestimate effect sizes (small-study bias) (34). The majority of trials lacked blinding of patients or outcome assessors, making subjective outcomes like pain scores prone to bias. High heterogeneity across studies—due to differences in block techniques, doses, and outcome measures—makes it difficult to pool meta-analysis results. Publication bias remains a concern, as funnel plots were not used in the cited meta-analyses and negative results may remain unpublished (45, 50). Additionally, most studies followed patients for only 72 h, which is insufficient to assess LB's effects on CPSP and long-term functional recovery.

## Reasons for contradictory meta-analysis findings

The contradictory conclusions between the meta-analyses can be attributed not only to differences in block technique composition but also to the timing of pain assessment (LB's theoretical advantage is at 24–72 h; assessment concentrated at 0-24 h may underestimate its effect), variations in rescue analgesia protocols (different thresholds and types of rescue analgesics affect comparability of opioid consumption), and surgical type (pain intensity and duration differ between VATS and thoracotomy; LB's benefit may be more pronounced in VATS).

## Recommendations for clinical practice

Based on current evidence, we offer the following recommendations for clinical practice. LB should not be used routinely for all thoracic surgeries; it should be selectively used for patients expected to have severe postoperative pain or who require early discharge or early mobilization. Given the more consistent evidence for SAPB, ESPB, or TPVB compared with ICNB, these techniques may be preferred when LB is used, although direct comparative evidence is lacking. LB should be part of a multimodal analgesia regimen, not as a sole analgesic method, and its use should not lead to reduction of other analgesic measures (e.g., acetaminophen, NSAIDs). For units already using continuous infusion pumps, the cost-effectiveness of LB may be less favorable.

## Priorities for future research

Future studies should prioritize the following questions. Head-to-head RCTs comparing LB efficacy across different block techniques are needed to determine the optimal combination. Factorial design RCTs of LB combined with different ERAS components would help isolate LB's independent contribution. RCTs with long-term follow-up (≥6 months) are required to confirm LB's sustained impact on CPSP. Optimal dosing studies for LB are lacking; most studies used fixed doses (133–266 mg), and whether a dose-response relationship exists is unclear. Health economic evaluations should incorporate indirect costs (e.g., patient productivity loss), not just direct medical costs. Finally, patient-reported outcomes such as satisfaction, quality of life, and functional status should be routinely assessed (35).
